# Recombinant p35 from Bacteria Can Form Interleukin (IL-)12, but Not IL-35

**DOI:** 10.1371/journal.pone.0107990

**Published:** 2014-09-26

**Authors:** Samadhi Aparicio-Siegmund, Jens M. Moll, Juliane Lokau, Melanie Grusdat, Jutta Schröder, Svenja Plöhn, Stefan Rose-John, Joachim Grötzinger, Philipp A. Lang, Jürgen Scheller, Christoph Garbers

**Affiliations:** 1 Institute of Biochemistry and Molecular Biology II, Medical Faculty, Heinrich-Heine University, Düsseldorf, Germany; 2 Institute of Biochemistry, Christian-Albrechts-University, Kiel, Germany; 3 Department of Gastroenterology, Hepatology, and Infectious Diseases, Heinrich-Heine-University, Düsseldorf, Germany; 4 Department of Molecular Medicine II, Heinrich-Heine University, Düsseldorf, Germany; Université Libre de Bruxelles, Belgium

## Abstract

The Interleukin (IL)-12 family contains several heterodimeric composite cytokines which share subunits among each other. IL-12 consists of the subunits p40 (shared with IL-23) and p35. p35 is shared with the composite cytokine IL-35 which comprises of the p35/EBI3 heterodimer (EBI3 shared with IL-27). IL-35 signals via homo- or heterodimers of IL-12Rβ2, gp130 and WSX-1, which are shared with IL-12 and IL-27 receptor complexes, respectively. p35 was efficiently secreted in complex with p40 as IL-12 but not with EBI3 as IL-35 in several transfected cell lines tested which complicates the analysis of IL-35 signal transduction. p35 and p40 but not p35 and EBI3 form an inter-chain disulfide bridge. Mutation of the responsible cysteine residue (p40_C197A_) reduced IL-12 formation and activity only slightly. Importantly, the p40_C197A_ mutation prevented the formation of antagonistic p40 homodimers which enabled the *in vitro* reconstitution of biologically active IL-12 with p35 produced in bacteria (p35_bac_). Reconstitution of IL-35 with p35_bac_ and EBI3 did, however, fail to induce signal transduction in Ba/F3 cells expressing IL-12Rβ2 and gp130. In summary, we describe the *in vitro* reconstitution of IL-12, but fail to produce recombinant IL-35 by this novel approach.

## Introduction

Cytokines were grouped into distinct families, mostly upon structural features and not upon homology among the amino acid sequences. These features include the protein fold or the usage of certain membrane-bound cytokine β-receptors, which are needed for signal transduction [1], [2]. Members of the IL-6 and IL-12 families have pleiotropic functions and are critically involved in proliferation, apoptosis and differentiation of T cells. Interestingly, members of both families share cytokine subunits as well as cellular receptors, suggesting a yet only poorly understood cross-talk between IL-6 and IL-12 type cytokines [1].

**Figure 1:**
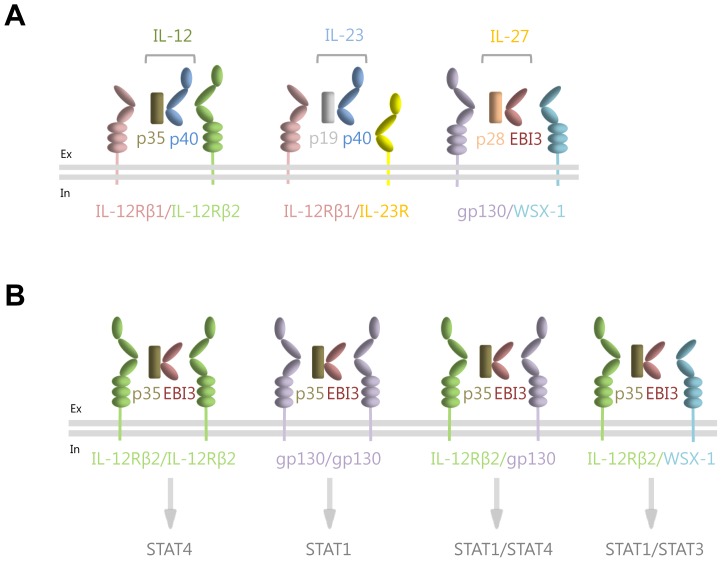
Schematic overview of the IL-12 family of cytokines. (**A**) Interleukin-12 (consisting of the p35 and the p40 subunits) signals via a heterodimer of the two β-receptors IL-12Rβ1 and IL-12Rβ2. Interleukin-23 (consisting of the p19 and the p40 subunits) signals via a heterodimer of the two β-receptors IL-12Rβ1 and the unique IL-23R. Interleukin-27 (consisting of its subunits p28 and EBI3) engages signaling via gp130 and the unique β-receptor WSX-1. (**B**) Interleukin-35 (consisting of its subunits p35 and EBI3) is able to signal via four different combinations of β-receptors, resulting in the phosphorylation and thus activation of different STAT proteins. Binding of IL-35 to an IL-12Rβ2 homodimer induces the activation of STAT4 homodimers [10], binding to a gp130 homodimer activates STAT1 homodimers [10], binding to an IL-12Rβ2/gp130 heterodimer induces STAT1/STAT4 heterodimerization [10], and binding of IL-35 to a IL-12Rβ2/WSX-1 heterodimer induces the activation of STAT1 and STAT3 [12].

The IL-12 family members IL-12 and IL-23 are secreted as composite cytokines, in which the cytokine subunit p35 and p19 are connected by an intra-chain disulfide bridge with the soluble α-receptor p40. In the absence of p35 or p19, p40 forms the antagonistic disulfide-connected homodimer p80 [3], [4], [5], [6], [7]. IL-12 engages a heterodimeric receptor complex of IL-12Rβ1/IL-12Rβ2 [8] (Figure 1A). IL-23 shares the IL-12Rβ1 with IL-12, but uses a unique IL-23R for signaling [9] (Figure 1A). The recently discovered IL-35 consists of p35 and EBI3 shared with IL-12 and IL-27, respectively, and signals via four different receptor complexes IL-12Rβ2/gp130, IL-12Rβ2/IL-12Rβ2, gp130/gp130 and IL-12Rβ2/WSX-1 [10], [11], [12] (Figure 1B). IL-27 (p28/EBI3) is traditionally grouped into the IL-6 family, since it uses gp130 for signaling, but has also been assigned to the IL-12 family [13], [14] (Figure 1A).

The IL-6 cytokine family consists of IL-6, IL-11, IL-27, IL-30, IL-31, leukemia inhibitory factor (LIF), oncostatin M (OSM), ciliary neurotrophic factor (CNTF), cardiotrophin-1 (CT-1), cardiotrophin-like cytokine (CLC) and neuropoeitin [1], [15], [16]. With the exception of IL-31, which signals through a heterodimer of GPL and OSMR, all IL-6 type cytokines engage at least one molecule of the ubiquitously expressed β-receptor glycoprotein 130 (gp130). gp130 can form homodimers (IL-6, IL-11, IL-30 [15], [17]) or heterodimers with WSX-1 (IL-27), LIFR (LIF, OSM, CNTF, CT-1, CLC) or OSMR (OSM). Cellular specificity is gained through additional membrane-bound or soluble non-signaling alpha receptors, which are used by several cytokines, such as IL-6R and soluble IL-6R (IL-6, IL-30), IL-11R (IL-11), CNTFR (CNTF, CLC) and soluble EBI3 (IL-27) [1].

IL-12 and IL-23 have been shown to activate mainly STAT3 but also STAT1, 4 and 5 [9], whereas IL-27 predominantly activates STAT1 and only to a lesser degree STAT3 and STAT5 [13]. Interestingly, IL-35 activates solely STAT1, but not STAT3, when signaling through a gp130 homodimer. However, a recent paper showed activation of STAT1 and STAT3 by IL-35 through a IL-12Rβ2/WSX-1 heterodimer [12]. IL-35 signaling through IL-12Rβ2/IL-12Rβ2 leads, however, to STAT4 phosphorylation, whereas IL-12Rβ2/gp130 induces STAT1 and STAT4 phosphorylation [10]. It is, however, completely unknown how the IL-35-induced STAT1/3/4 activation pattern is executed and regulated on the receptor level.

Here, we show that IL-35, in contrast to IL-12 and IL-23, is not efficiently secreted from transfected cells, making the biochemical characterization of IL-35 signaling impossible. To enable the analysis of IL-35 signal transduction, we develop a protocol to purify recombinant biologically active p35_bac_ from *E.coli*. Combining p35_bac_ with p40_C197A_, a solely monomeric form of p40, resulted in biologically active IL-12 that induced STAT1 and STAT3 phosphorylation as well as cytokine-dependent cellular proliferation. In contrast to this, recombinant p35_bac_ did not form biologically active IL-35 when combined with EBI3.

## Materials and Methods

### Cells and Reagents

Ba/F3-gp130 cells have been described previously [18]. HEK293 and COS7 cells were obtained from DSMZ (Braunschweig, Germany). All cells used in this study were grown in DMEM high glucose culture medium (Gibco, Life Technologies, Grand Island, NY, USA) supplemented with 10% fetal bovine serum, penicillin (60 mg/l) and streptomycin (100 mg/l) at 37°C in an incubator with 5% CO_2_ in a water-saturated atmosphere. Ba/F3-gp130 cells were cultured using 10 ng/ml recombinant Hyper-IL-6 (fusion of IL-6 and the sIL-6R with a peptide linker) which was expressed and purified as described previously [19], [20]. The Ba/F3-gp130-IL-12Rβ1-IL12-Rβ2 cells were cultured in the presence of 0.2% conditioned Hyper-IL-12 supernatant obtained from transfected HEK293 cells. The anti-hIL-6R monoclonal antibody 4-11 was described previously [21]. Antibodies against Myc (71D10), STAT3 (124H6), pSTAT3 Y705 (D3A7), STAT1 and pSTAT1 Y701 (58D6) were from Cell signaling. Anti-FLAG antibody was from Sigma-Aldrich. Anti-human-IgG, anti-rabbit-IgG, and anti-mouse-IgG antibodies were purchased from Thermo Scientific.

### Plasmids

Expression plasmids for Hyper-IL-6, Hyper-IL-27 and Hyper-IL-30 have been described previously [17]. To clone the Hyper-IL-35 cDNA the pcEP-PU plasmid coding for Hyper-IL-27 was used as a template. Hyper-IL-27 consists of a signal peptide (METDTLLLWVLLLWVPGSTGD), mEBI3, a linker peptide (VPGVGVPGVG), mp28 and a myc- and His-tag. To insert a *Sma*I restriction site between linker and p28 by site directed mutagenesis this vector was digested with *Afl*II and *Not*I and the sticky ends were filled up using Klenow fragment (Thermo Scientific, Schwerte, Germany) in order to subclone the fragment by blunt end ligation into the pCR-Script vector.

The following primers were used to perform standard protocol mutagenesis PCR: SmaI-1: TTCACCCTCAGGAACTCGAAACCCCATGCC, SmaI-2: TGGGAAGCCCACCCCGGGTAC CCCTACTCC, SmaI-3: GGAGTAGGGGTACCCGGGGTGGGCTTCCCA, SmaI-4: AGTGGCGGC CCTCGAGCCGCGGCCGCAGAA. The resulting mutated fragment was cloned into pcR-Script-IL-27 by digestion of both with the restriction enzymes *Bsu*36I and *Xho*I. p35 was amplified from pcDNA3.1-FLAG-p35-His with p35-Fwd: GGGGTGAGGGTCATTCCAGTCTCTGGA and p35-Rev: GCGGCTCGAGGGGCGGAGC TCAGATA inserting a *Xho*I site in 3′ of p35. p28 was excised from pcR-Script by *Sma*I and *Xho*I and replaced by p35. The resulting IL-35 cDNA was subcloned into pcEP-PU through *Hin*dIII and *Xho*I.

To test the influence of the linker on the secretion of overexpressed IL-35 we exchanged it by a 3xGGGGS-linker. To achieve this the double stranded oligonucleotide coding for the GGGGS-linker was inserted into pcR-Script-IL-35 through *Bam*HI and *Sma*I excising EBI3 and the former linker. EBI was then amplified by PCR (Fwd-Primer: GATCAAGCTTTATGGAGACAGACACACTCC, Rev-Primer: GATCGGATCCCTTATGGGGTGCACTTT) inserting a *Hin*dIII site in 5′ and a *Bam*HI site in 3′ of EBI3 and then cloned into the vector containing the GGGGS-linker and p35 through these restriction enzymes. The resulting cDNA was subloned into pcEP-PU through *Hin*dIII and *Xho*I. The cDNA coding for Hyper-IL-12 was cloned in analogy into pcEP-PU to generate a Hyper-IL-12 with myc and his-tag.

To clone the C92A mutant of p35 the following primers were used for PCR site directed mutagenesis: p35-AflII-Fwd: TAAACTTAAGAGGGTCATTC, C92A-Fwd: ACGAGAGTGCCCTGGCTACT, C92A-Rev: AGTAGCCAGGGCACTCTCGT, p35-NotI-Rev: ATGTGCGGCCGCGGCGGAGC. The resulting fragment was ligated into pcDNA3.1 through *Afl*II and *Not*I.

WT and C92A p35 were subcloned into pET-23a for bacterial expression by amplifying them with the following primers from pcDNA3.1: p35-NdeI-Fwd: GATCCATATGAGGGTCATTCCAGTCTC TGG, p35-NotI-Rev: ATGTGCGGCCGCGGCGGAGC and using *Nde*I and *Not*I for digestion.

### Expression, purification, and renaturation of murine p35_bac_

For gene expression 1 l of LB medium supplemented with 100 µg/ml ampicillin was inoculated from an overnight starter culture and grown to on optical density at 600 nm (OD_600_) of 0.6 at 37°C. Protein production was induced by addition of 0.1 mM isopropyl 1-thio-β-galactopyranoside. Following incubation at 37°C for additional 4 h bacteria were harvested by centrifugation (4000×g, 4°C). Cell pellets were resuspended in lysis buffer (50 mM Tris-HCl, pH 8.0 containing 6 M guanidine hydrochloride and 10 mM imidazole). Cell lysis was carried out by sonification (Bandelin Sonoplus HD70). Lysates were cleared by centrifugation (10000×g, 20 min, 4°C) and supernatants loaded on a 1 ml HisTrap HP column (GE Healthcare) equilibrated with lysis buffer. Bound proteins were eluted with lysis buffer containing 500 mM imidazole. Imidazole was removed from mp35*_bac_* samples by ultrafiltration using Amicon Ultra-15 filters (Millipore) with a 10,000 Da molecular weight cut off. Denatured mp35*_bac_* was refolded at a concentration of 1 mg/ml by dialysis (4°C) against 50 mM Tris-HCl (pH 8.0) containing 250 mM NaCl. Dialysed mp35*_bac_* was cleared by centrifugation (1000×g, 20 min, 4°C), concentrated by ultrafiltration and applied to a Superdex 75 10/300 GL (GE Healthcare) column connected to an Äkta Purifier 10 system. Fractions containing mp35*_bac_* were concentrated by ultrafiltration. Purified proteins were analyzed by SDS-PAGE.

### Purification of CD4+ T cells

6-8 weeks old C57/BL6J mice were obtained from Jackson Laboratories (Bar Harbor, Maine, USA). CD4^+^ T cells were enriched by positive selection using the CD4 (L3T4) MicroBeads (Miltenyi) from single cell suspended splenocytes and lymphnode cells following the manufacturer's instructions.

1×10^5^ cells per well were cultured on plates coated with 0.5 µg/ml anti-CD3 (eBioscience) and 2 µg/ml soluble anti-CD28 (BD Pharmingen). 10% of the indicated cell culture supernatant was added and supernatant was harvested after three days to assess the concentration of IFN-γ.

### IFN-γ ELISA

For detection of IFN-γ concentrations in cell culture supernatants the Mouse IFN-γ DouSet ELISA Development System from R&D Systems was used according to the manufacturer's instructions. Supernatants were diluted to the appropriate concentrations in reagent diluent. For the development the BM Blue POD Substrate solution from Roche was applied and the reaction was stopped using 1.8 M H_2_SO_4_. The absorption was measured at a wavelength of 450 nm in the Tecan infinite M200 PRO reader.

### Cytokine stimulation of cells and cell lysis

For cytokine stimulation Ba/F3-gp130 or Ba/F3-gp130-IL-12Rβ1-IL-12Rβ2 cells were washed twice in PBS and subsequently starved for 3 h in serum-free DMEM. Conditioned supernatants were preincubated with recombinant p35 variants for 30 min at 37°C and added to 2×10^6^ cells at the concentrations indicated. The cells were stimulated for 15 min at 37°C, collected by centrifugation, and the pellet was lyzed in 60 µl of 2.5 x Laemmli-Buffer.

### Pulldown assays

To assess binding of the different cytokine subunits to each other, COS7 cells were transiently transfected using TurboFect transfection reagent (Thermo Scientific, Schwerte, Germany) according to the manufacturer's instructions. After 48 h cells were washed in PBS and lyzed in 250 µl of immunoprecipitation buffer (20 mM Tris, pH 7.5, 150 mM NaCl, 1 mM EDTA, 1 mM EGTA, 1% Triton X-100, 2.5 mM sodium pyrophosphate, 1 mM β-glycerophosphate, 1 mM sodium orthovanadate and Complete Protease Inhibitor Cocktail Tablets (Roche, Mannheim, Germany)) for 1 h at 4°C. The lysates were centrifuged at 1000 rpm for 3 min and 100 µl of each of the supernatants were mixed and incubated overnight at 4°C. After adding 75 µl of Protein-A-agarose beads (Thermo Scientific, Schwerte, Germany) and incubation for 4 h at 4°C beads were spun down at 1000 rpm for 5 min at 4°C and 10% of the supernatant was used for Western blotting. The beads were washed five times with 500 µl immunoprecipitation buffer, boiled in 50 µl 1 x Laemmli Buffer and 50% were loaded on a 15% SDS-PAGE gel.

### SDS-PAGE and Western Blotting

Lysates of COS7 cells with overexpressed cytokine subunits were prepared by suspending the cells in 250 µl of mild lysis buffer (50 mM Tris (pH 7.5), 150 mM NaCl, 1% Triton X-100 and Complete Protease Inhibitor Cocktail Tablets (Roche, Mannheim, Germany)), incubating them for 30 min on ice, centrifuging at 13,000 rpm for 5 min at 4°C and transferring the supernatant to a new tube. Total protein concentration was determined by applying the BCA protein assay kit (Thermo Scientific, Schwerte, Germany). 50 µg of total protein was separated by SDS-PAGE. Semi-dry Western blotting was performed using the Trans-Blot Turbo Transfer System from Bio-Rad (München, Germany).

### Proliferation assays

Proliferation of the different Ba/F3-gp130 cell lines was determined as described previously [22] using the Cell Titer Blue Cell viability assay reagent (Promega, Karlsruhe, Germany) following the manufacturer's protocol. The extinction was measured using a Tecan infinite M200 PRO reader (excitation 560 nm, emission 590 nm, gain 90, i-control 1.7 software, Tecan AG, Maennedorf, Switzerland). Normalization of relative light units (RLU) was achieved by subtraction of negative control values. All values were measured in triplicates per experiment.

## Results

### Single-chain Hyper-IL-6, Hyper-IL-12, Hyper-IL-27 and Hyper-IL-30, but not Hyper-IL-35 were efficiently secreted and biologically active

**Figure 2:**
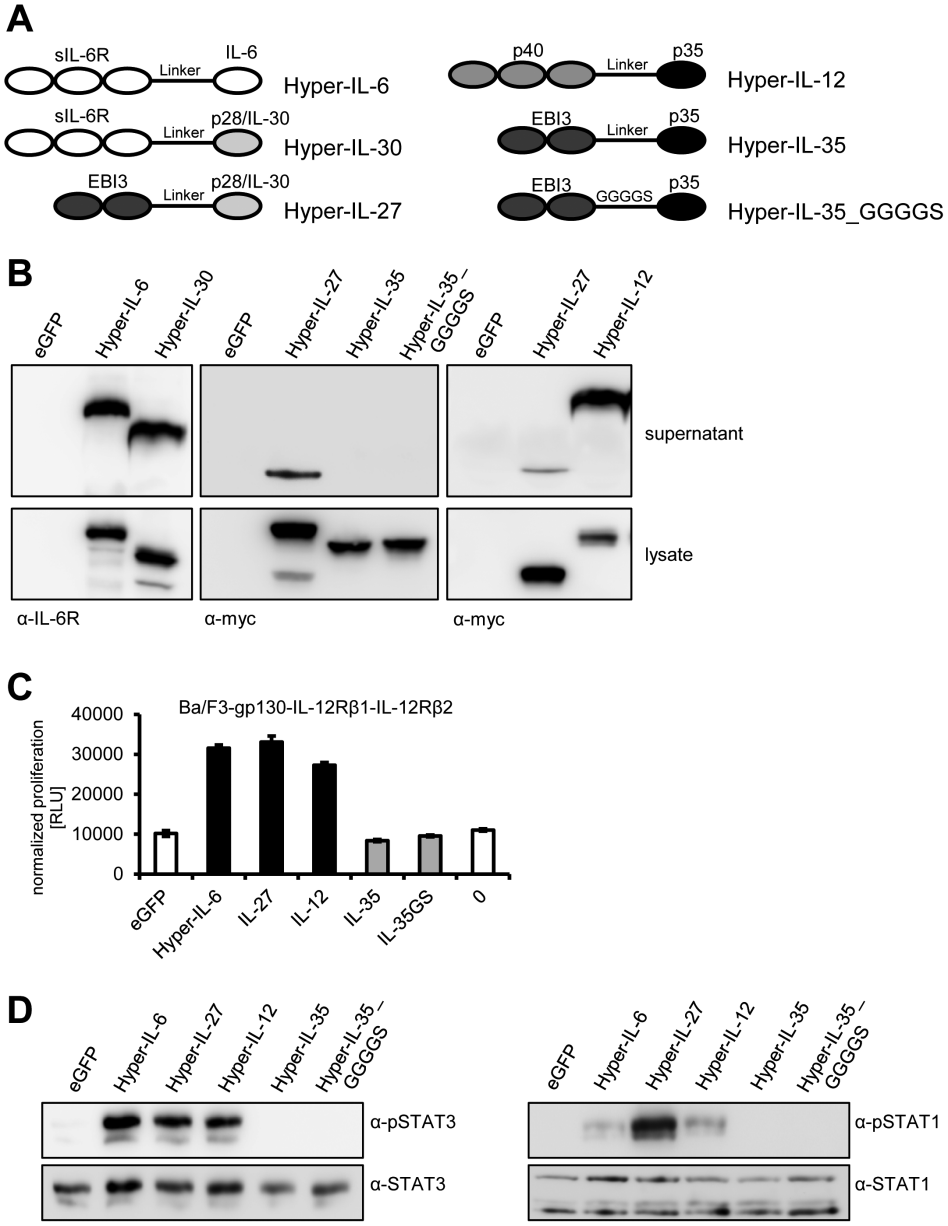
In contrast to other Hyper-cytokines, Hyper-IL-35 is not efficiently secreted from cells. (**A**) Schematic representation of the six different Hyper-constructs used in this study. In Hyper-IL-6, the extracellular domains of the IL-6R are fused to IL-6, whereas in Hyper-IL-30 p28/IL-30 replaces IL-6. Hyper-IL-27 represents a composite cytokine where EBI3 is fused via a flexible linker to p28/IL-30. Hyper-IL-12 depicts p40 fused to p35, and Hyper-IL-35 is a linker-based fusion-protein of EBI3 and p35. All Hyper-cytokines contain a C-terminal myc-tag with the exception of Hyper-IL-6 and Hyper-IL-30, which are untagged. (**B**) HEK293 cells were transiently transfected with the different constructs shown in panel (A) or a control plasmid containing eGFP. Supernatant was taken 48 h post transfection. Cells were lysed, and expression and secretion of the different Hyper-cytokines was assessed by Western blotting with monoclonal antibodies against the IL-6R (for the detection of Hyper-IL-6 and Hyper-IL-30) or the myc-tag (for the detection of Hyper-IL-27, Hyper-IL-35, Hyper-IL-35_GGGGS and Hyper-IL-12). Western blots shown are representative of three different experiments with similar outcomes. (**C**) Equal amounts of Ba/F3-IL-12Rβ1-IL-12Rβ2 cells were incubated with 10% of supernatants derived from HEK293 cells described in panel (B). As a further negative control, cells were incubated without the addition of supernatant (right column). Cellular proliferation was determined 48 h later as described in *Materials and Methods*. (**D**) Equal amounts of Ba/F3-IL-12Rβ1-IL-12Rβ2 cells were stimulated with supernatants from HEK293 cells transiently transfected with the constructs indicated above the Western blots for 15 min. Phosphorylation of STAT3 and STAT1 was determined per Western blotting. Total amounts of STAT1 and STAT3 were visualized as internal loading control. The Western blots shown are representative of three different experiments with similar outcomes, and the proliferation assay was measured in triplicates and is representative out two performed experiments.

Heterodimeric cytokines can be genetically fused with a peptide linker. These so-called single-chain or ‘Hyper’-cytokines have a higher activity compared to the unlinked cytokine subunits, making them an ideal tool for the biochemical characterization of cytokine signal transduction [19], [23]. Here, we compared five different Hyper-cytokines Hyper-IL-6 (hIL-6/shIL-6R) [19], Hyper-IL-30 (mp28/shIL-6R) [17], Hyper-IL-27 (mp28/mEBI3) [23], Hyper-IL-12 (mp35/mp40) [24], and Hyper-IL-35 (mp35/mEBI3) [11] covering essential members of the IL-6 and IL-12 cytokine families (Figure 2A) with respect to secretion and biological activity. All Hyper-cytokines contained their original signal peptide of the respective soluble alpha-receptor subunit. The Hyper-cytokines were detected in the lysates and, with the exception of IL-35, in the cell culture supernatant of transiently transfected HEK293 cells by Western blotting (Figure 2B). To exclude that the standard linker peptide prevents secretion of Hyper-IL-35, we created a second IL-35 cDNA, which codes for a 3xGGGGS-linker between EBI3 and p35 [25]. Hyper-IL-35_GGGGS was expressed in similar amounts as all other Hyper-cytokines in transiently transfected HEK293 cells (Figure 2B), but it was also not secreted into the cell culture supernatant. To exclude a cell-specific effect, HeLa, COS7 and CHO cells were transfected with Hyper-IL-12, Hyper-IL-27 and Hyper-IL-35 cDNAs which then secreted Hyper-IL-12 and Hyper-IL-27 but not IL-35 (data not shown).

The biological activity of the Hyper-cytokines was verified using Ba/F3-gp130-IL-12Rβ1-IL-12Rβ2 cells. Ba/F3 is a murine pre-B cell-line that proliferates solely in response to IL-3. Ba/F3 cells express WSX-1 but lack expression of gp130, IL-12Rβ1 and IL-12Rβ2. Stable transduction of Ba/F3-cells with cDNAs coding for cytokine receptors such as gp130, IL-12Rβ1 or IL-12Rβ2 extended their cytokine responsiveness towards IL-6/sIL-6R, IL-27 or IL-12. As shown in Figure 2C, conditioned supernatants containing Hyper-IL-6, Hyper-IL-12 and Hyper-IL-27 induced cellular proliferation of Ba/F3-gp130-IL-12Rβ1-IL-12Rβ2 cells, whereas supernatant from eGFP transfected cells or no addition of conditioned supernatant did not induce cellular proliferation. In contrast to Hyper-IL-6, Hyper-IL-12 and Hyper-IL-27, supernatants from Hyper-IL-35 transfected cells did not induce Ba/F3-gp130-IL-12Rβ-IL-12Rβ2 cell proliferation, which was most likely due to compromised Hyper-IL-35 secretion. It was, however, not possible to use intracellular Hyper-cytokines from cellular lysates to induce Ba/F3-gp130-IL-12Rβ1-IL-12Rβ2 cell proliferation (data not shown). We have to note that it is unknown if Ba/F3-gp130-IL-12Rβ1-IL-12Rβ2 cells will also grow in the dependence of IL-35, albeit the receptor composition should be sufficient to signal via IL-35 [8], [10], [26]. IL-35 has been shown to activate STAT1, but not STAT3, a property that has not been reported for any other cytokine that signals through gp130 [1], [10]. In contrast, a recent paper described activation of STAT3 through the gp130/WSX-1 heterodimer in B cells [26]. Ba/F3-gp130-IL-12Rβ1-IL-12Rβ2 cells express WSX-1 endogenously, and thus comprise all possible receptors that have been published for IL-35. We detected phosphorylation of STAT1 and STAT3 when cells were stimulated with Hyper-IL-6 (activating gp130/gp130), Hyper-IL-27 (activating gp130/WSX-1) and Hyper-IL-12 (activating IL-12Rβ1/IL-12Rβ2), but neither of them when cells were stimulated with Hyper-IL-35 supernatants (Figure 2D).

### The heterodimeric cytokines IL-12 and IL-27, but not IL-35, were secreted and biologically active

**Figure 3:**
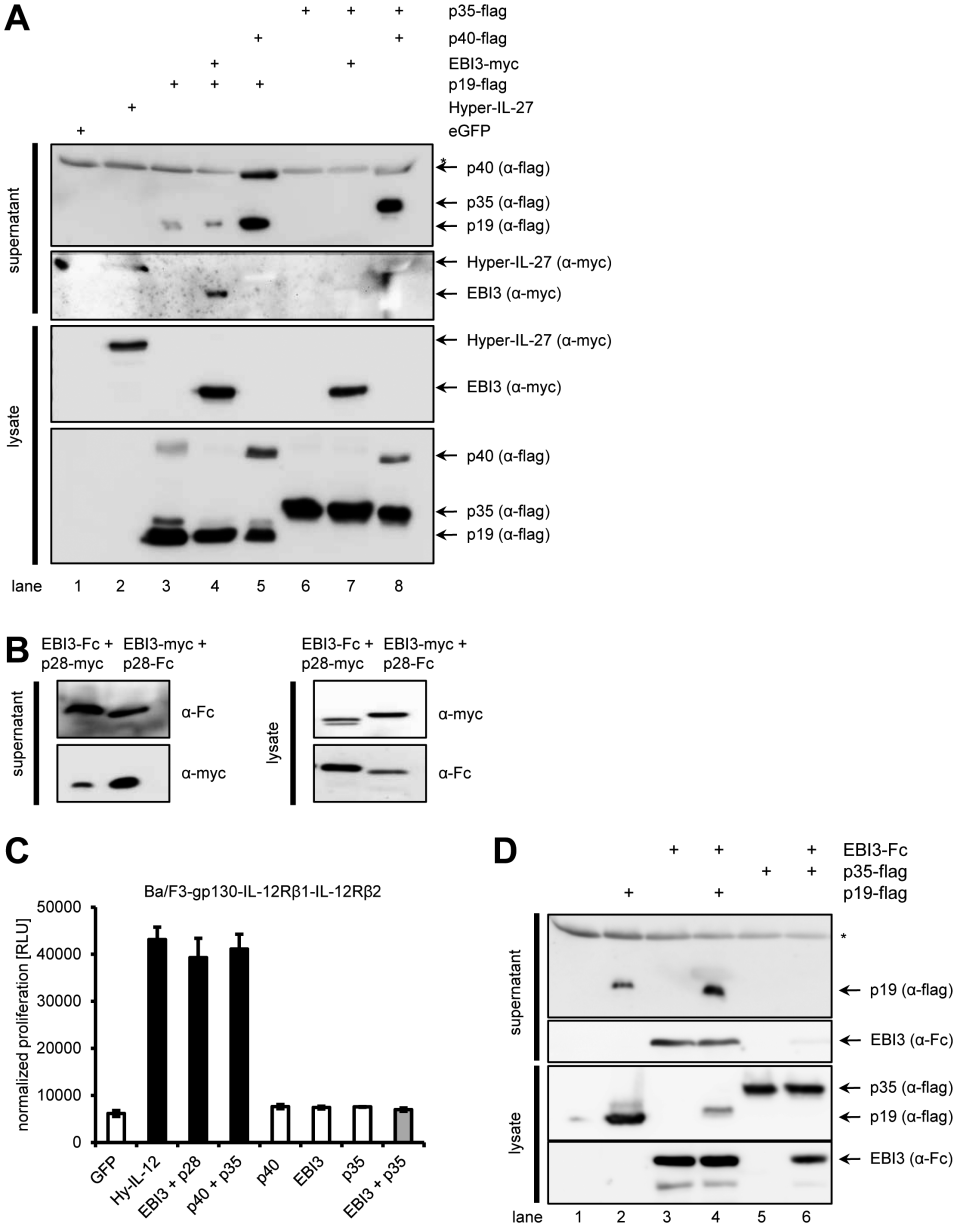
p28 and EBI3 (IL-27), p40 and p35 (IL-12) as well as p40 and p19 (IL-23) form heterodimeric cytokines and are efficiently secreted, but not EBI3 and p35 (IL-35). (**A**) HEK293 cells were transiently transfected with plasmids encoding the constructs indicated above the Western blots. Supernatant was taken 48 h post transfection. Cells were lysed, and expression and secretion of the different cytokines was assessed by Western blotting with monoclonal antibodies against the flag- or the myc-tag. Western blots shown are representative of three different experiments with similar outcomes. The asterisk denotes an unspecific band detected by the flag-antibody. (**B**) HEK293 cells were transiently transfected with plasmids containing either EBI3-Fc and p28-myc or EBI3-myc and p28-Fc. Expression and secretion of the respective proteins was analyzed by Western blotting as described in panel (A) with antibodies directed against the Fc- or the myc-tag. (**C**) Equal amounts of Ba/F3-IL-12Rβ1-IL-12Rβ2 cells were incubated with 10% of supernatants derived from transfected cells described in panel (A) and (B). Cellular proliferation was determined 48 h later as described in *Materials and Methods*. (**D**) HEK293 cells were transiently transfected with plasmids encoding the constructs indicated above the Western blots. Supernatant was taken 48 h post transfection. Cells were lysed, and expression and secretion of the different cytokines was assessed by Western blotting with monoclonal antibodies against the flag- or the Fc-tag. Western blots shown are representative of three different experiments with similar outcomes. The asterisk denotes an unspecific band detected by the flag-antibody.

To test if the protein fusion of mp35 and mEBI3 prevented secretion of biologically active IL-35, we sought to reconstitute heterodimeric IL-35 by co-transfection of two separate cDNAs coding for mp35 and mEBI3 (Figure 3A). First, we tested the ability of the different subunits to form the other heterodimeric cytokines IL-12, IL-23 and IL-27. Co-transfection of mp19/mp40 (Figure 3A, lane 5), mp35/mp40 (Figure 3A, lane 8) and mp28/mEBI3 (Figure 3B) cDNAs in HEK293 cells resulted in the secretion of IL-12, IL-23 and IL-27 as shown by Western blotting of cell lysates and cell culture supernatants. Biological activity of the secreted composite cytokines IL-12 and IL-27 was verified using Ba/F3-gp130-IL-12Rβ1-IL-12Rβ2 cells (Figure 3C), demonstrating that the IL-35 subunits p35 and EBI3 were principally able to form biologically active composite cytokines IL-12 and IL-27. Supernatant from eGFP transfected cells or cells solely transfected with the p40 subunit or the p35 subunit did not induce proliferation of Ba/F3-gp130-IL-12Rβ1-IL-12Rβ2 cells (Figure 3C). P19 alone was only poorly and p35 was not secreted at all from human cells in the absence of p40 (Figure 3A, lanes 3 and 6, and [14], [27]). Co-expression of p35 with EBI3 did not lead to secretion of p35 (IL-35) (Figure 3A, lane 7) and the supernatant did not induce proliferation of Ba/F3-gp130-IL-12Rβ1-IL-12Rβ2 cells (Figure 3C). Interestingly, co-transfection of EBI3 with p35 resulted in significantly reduced secretion of EBI3 (Figure 3D, lane 6), whereas transfection of either EBI3 alone (Figure 3D, lane 3) or EBI3 in combination with p19 (Figure 3D, lane 4) resulted in efficient secretion of EBI3. Again, p35 could not be detected in the cell culture supernatant when transfected alone (Figure 3D, lane 5) or in combination with EBI3 (Figure 3D, lane 6). These results suggest that EBI3 and p35 interact intracellularly, but cannot be efficiently secreted. These experiments confirmed our findings with the single-chain Hyper-IL-35 protein and are in line with previous findings that secretion of IL-35 is relatively poor, compared to that of IL-12 and IL-27 [2].

### p35 interacts with EBI3

**Figure 4:**
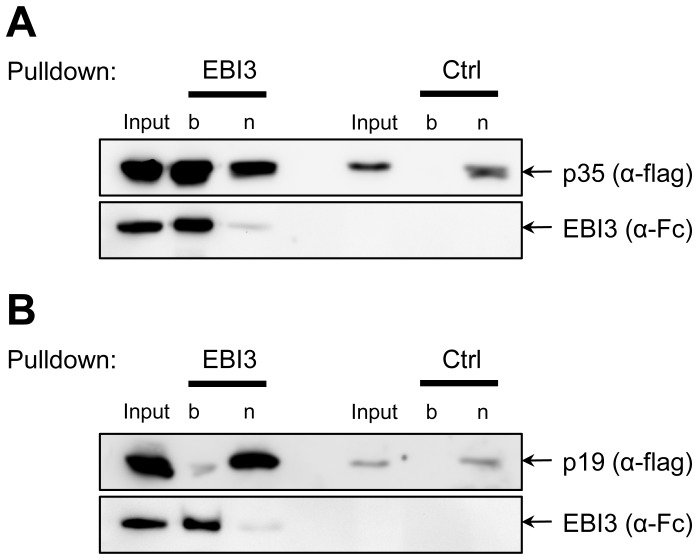
Interaction between p35 and EBI3. (**A**) HEK293 cells were transiently transfected with either p35 or EBI3. Cells were lysed, and lysates were mixed. To check for unspecific binding of p35 to the beads, lysates of p35-transfected cells without EBI were used as control (Ctrl). Pulldown was performed as described in *Materials and Methods*. Proteins were analyzed by Western blotting with antibodies against flag- and Fc-tag. (**B**) The experiment was performed as described under panel (A), but p19-transfected cells were used instead of p35. “b” denotes the bound fraction, “n” the non-bound proteins.

Next, we verified if p35 and EBI3 interacted with each other when expressed in mammalian cells via pulldown [2], [11], [25]. Fc-tagged EBI3, flag-tagged p35 and flag-tagged p19 were separately expressed in HEK293 cells. Since IL-35 was not secreted, cell lysates were used to check for interaction. The cell lysate containing EBI3 was mixed with lysate containing either p35 or p19 and incubated overnight to allow protein complex formation. Afterwards, EBI3 was precipitated with Protein-A-agarose beads via the Fc tag. As shown in Figure 4A, flag-tagged p35 was precipitated with Fc-tagged EBI3. Incubation of Protein-A-agarose beads with lysate containing only p35 did not reveal any p35 binding, demonstrating that p35 specifically interacted with EBI3. In contrast, EBI3 did not precipitate flag-tagged p19, underlining the specificity of the EBI3/p35 interaction (Figure 4B). Again, p19 alone did also not bind to the Protein-A-agarose beads in the absence of EBI3 (Figure 4B). These results show that the lack of secretion of heterodimeric IL-35 cannot be explained through impaired interaction of the two proteins within the cell. We cannot exclude that other, so far unknown, proteins are needed for IL-35 secretion that are not present in our *in vitro* setting, but facilitate IL-35 secretion *in vivo*.

### The two cysteine residues that form the inter-molecular disulfide bond between p40 and p35 are dispensable for IL-12 action

**Figure 5:**
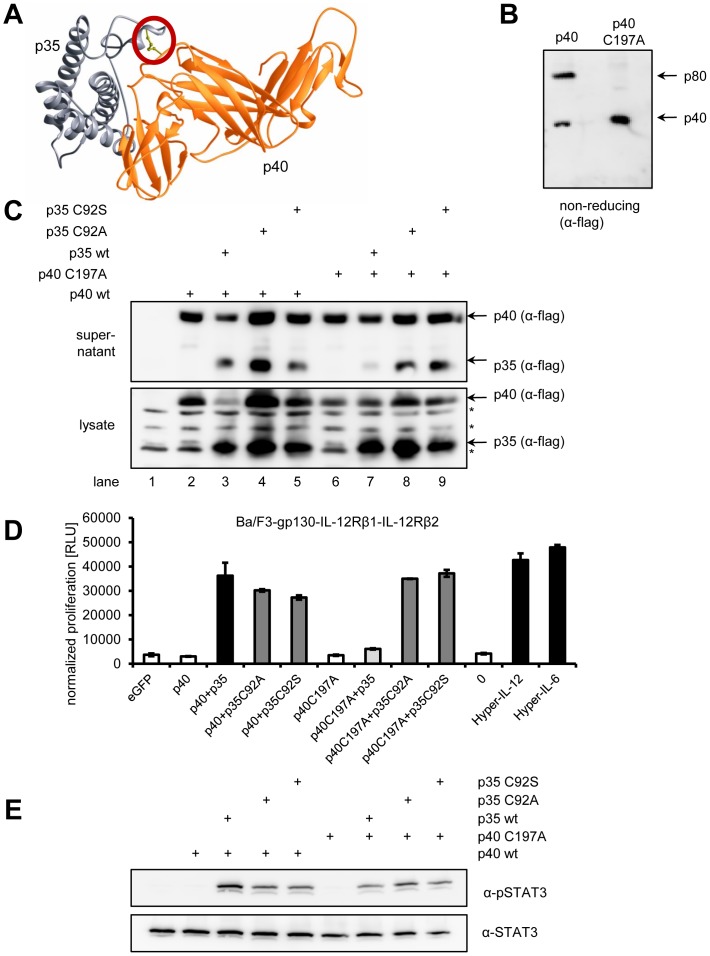
The disulfide bond p35C92-C197p40 is dispensable for the biological activity of IL-12. (**A**) Schematic representation of IL-12 comprising p35 (gray) and p40 (orange) according to [28]. The inter-molecular disulfide bond p35C92-C197p40 is highlighted with a red circle. (**B**) 20 µl conditioned supernatant of HEK293 cells transiently transfected with either p40 wildtype or p40C197A were separated by SDS-PAGE under non-reducing conditions and proteins visualized by Western blotting with a flag-specific antibody. (**C**) HEK293 cells were transiently transfected with plasmids encoding the constructs indicated above the Western blots. Supernatant was harvested 48 h post transfection. Cells were lysed, and expression and secretion of the different cytokines was assessed by Western blotting with monoclonal antibodies against the flag-tag. Unspecific bands detected by the flag-antibody are denoted with asterisks. (**D**) Ba/F3-IL-12Rβ1-IL-12Rβ2 cells were incubated with 10% of supernatants derived from HEK293 cells transiently transfected with the constructs given below the diagram. Cellular proliferation was determined 48 h later as described in *Materials and Methods*. (**E**) Equal amounts of Ba/F3-IL-12Rβ1-IL-12Rβ2 cells were stimulated with supernatants from HEK293 cells transiently transfected with the constructs indicated above the Western blots for 15 min. Phosphorylation of STAT3 was determined per Western blotting. Total amounts of STAT3 were visualized as internal loading control. The Western blots shown are representative of three different experiments with similar outcomes, and the proliferation assay was measured in triplicates and is representative of two performed experiments.

In contrast to IL-27, IL-12 is stabilized by an inter-chain disulfide bond between cysteine 92 of p35 and cysteine 197 of p40 (p35_C92_-p40_C197_) (Figure 5A and [28]). In human p35, the corresponding cysteine 74 forms an inter-chain disulfide bridge with cysteine 199 of p40. The p35_C74S_ mutant was still able to form complexes with p40 [28]. Consequently, Cys92 in p35 was mutated to alanine (p35_C92A_) or serine (p35_C92S_). In the absence of p35, IL-12p40 is secreted as an antagonistic disulfide-linked homodimer (p80) [4], [5], [6]. Gillessen et al. [4] have previously shown that treatment of p80 with reducing agents destroyed the p80 dimer and resulted in monomeric p40, suggesting that p80 is linked by an inter-chain disulfide bridge. To prevent p40 homo-dimerization, Cys197 of p40 was mutated to alanine (p40_C197A_). As expected, supernatant from transiently transfected HEK293 cells contained monomeric as well as dimeric p40 as demonstrated on a non-reducing gel (Figure 5B). Mutation of Cys197 to Ala in p40 prevented dimer formation, as p40_C197A_ was solely monomeric (Figure 5B). It was not known if this mutation still allows IL-12 formation. To answer this question, p35_C92A_, p35_C92S_ and p40_C197A_ were expressed in transiently transfected HEK293 cells and analyzed by Western blotting (Figure 5C). As shown before, transfection of p40 alone resulted in its secretion into the supernatant (Figure 5C, lane 2). When co-expressed with p35, p35_C92A_ or p35_C92S_, all three p35 variants were efficiently secreted in combination with p40 (Figure 5C, lane 3–5). Next, we tested the ability of p40_C197A_ to facilitate secretion of p35, p35_C92A_ and p35_C92S_. Whereas p40_C197A_ alone was again efficiently secreted (Figure 5C, lane 6), we could only detect small amounts of p35 in the supernatant when co-expressed with p40_C197A_ (Figure 5C, lane 7). In contrast, co-expression of p40_C197A_ in combination with either p35_C92A_ or p35_C92S_ led to secretion of both IL-12 subunits (Figure 5C, lane 8 and 9). We concluded from these results that the Cys92 of p35 and Cys197 of p40 were dispensable for IL-12 formation and secretion.

Next, we investigated the biological activity of the IL-12 disulfide-mutants using Ba/F3-gp130-IL-12Rβ1-IL-12Rβ2 cells. As positive controls, cell supernatant containing Hyper-IL-6, p40/p35 or Hyper-IL-12 induced robust proliferation of Ba/F3-gp130-IL-12Rβ1-IL-12-Rβ2 cells (Figure 5D, black bars). In line with their secretion, conditioned supernatants containing p40/p35_C92A_, p40/p35_C92S_ p40_C197A_/p35_C92A_ and p40_C197A_/p35_C92S_ induced proliferation of Ba/F3-gp130-IL-12Rβ1-IL-12-Rβ2 cells (Figure 5D, dark grey bars). Interestingly, we observed only a weak proliferation of Ba/F3-gp130-IL-12Rβ1-IL-12-Rβ2 cells when stimulated with supernatant containing p40_C197A_/p35 (Figure 5D, light gray bar). We concluded from this that although p35 in this combination was less efficiently secreted (Figure 5C, lane 7), the small amount of IL-12 was nevertheless biologically active and able to induce cellular proliferation. As negative controls, conditioned supernatant from cells transfected with eGFP, p40 or p40_C197A_ did not induce proliferation of Ba/F3-gp130-IL-12Rβ1-IL-12-Rβ2 cells (Figure 5D, unfilled bars).

Accordingly, conditioned supernatant containing p40/p35 induced phosphorylation of STAT3 which was completely absent when p40 was expressed alone (Figure 5E). p40 in combination with p35_C92A_ or p35_C92s_ showed a weaker, but consistent STAT3 phosphorylation. Furthermore, p40_C197A_/p35_C92A_ and p40_C197A_/p35_C92S_ induced STAT3 phosphorylation which correlated with Ba/F3 cell proliferation (Figure 5D, 5E). Supernatant containing p40_C197A_/p35, which only induced a very weak proliferative response (Figure 5D), displayed in line with this only a slight phosphorylation of STAT3 (Figure 5E).

Taken together, these data show that the inter-chain disulfide bridge of p35 and p40 is dispensable for secretion and biological activity of IL-12.

### *In vitro* reconstituted IL-12 composed of recombinant p35 from bacteria and p40_C192A_ from HEK293 cells is biologically active

Since we did not obtain secreted p35 and IL-35 from cell culture supernatants of transfected mammalian cells, we decided to express p35 in bacteria and to combine this protein with p40 and EBI3-conditioned supernatant to *in vitro* reconstitute IL-12 and IL-35, respectively. Previously, we combined recombinant p28 from bacteria with conditioned cell culture supernatant containing EBI3 which led to *in vitro* reconstitution of IL-27 [17].

**Figure 6:**
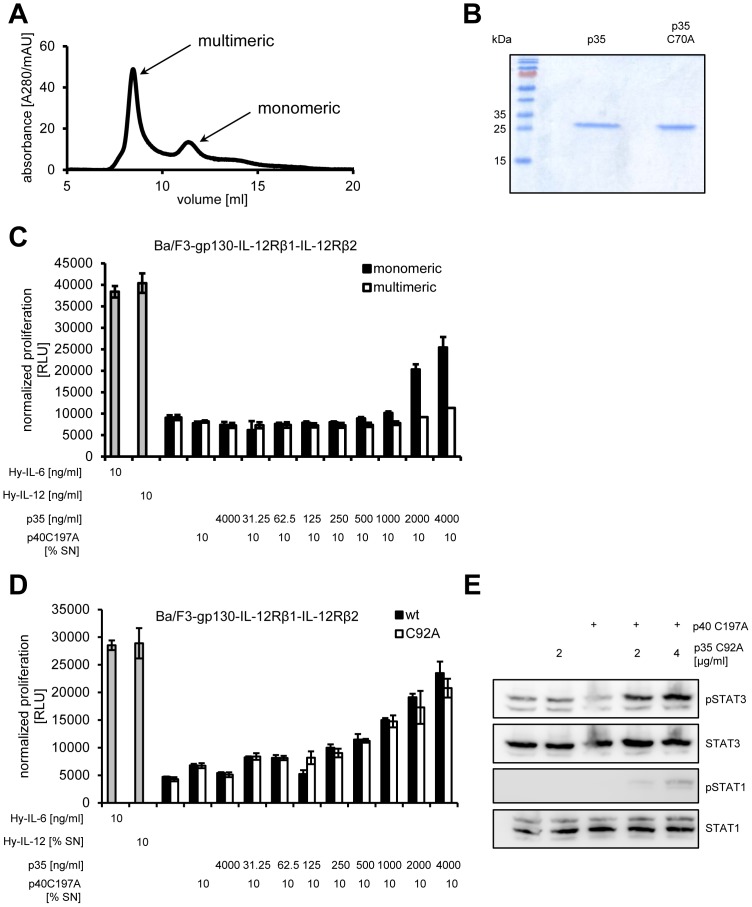
Recombinant p35 from bacteria forms biologically active IL-12. (**A**) Refolded p35_bac_ and p35_bac/C92A_ were subjected to size exclusion chromatography. Monomeric protein was separated from multimeric assemblies on a Superdex 75 10/300 GL equilibrated in 50 mM Tris-HCl (pH 8.0) containing 250 mM NaCl. (**B**) Purity of the bacterial produced p35_bac_ and p35_bac/C92A_ was analyzed by SDS-PAGE on a reducing gel via Coomassie brilliant blue staining. 5 µg protein were loaded per lane. (**C**) Equal amounts of Ba/F3-IL-12Rβ1-IL-12Rβ2 cells were incubated with 10 ng/ml Hyper-IL-6, 10% conditioned supernatant containing Hyper-IL-12, or 10% conditioned supernatant containing p40_C197A_ with increasing amounts of either monomeric or multimeric p35. (**D**) Equal amounts of Ba/F3-IL-12Rβ1-IL-12Rβ2 cells were treated as described under panel (C), but with either recombinant p35_bac_ or p35_bac/C92A_ (0–4000 ng/ml). Cellular proliferation in both experiments was determined 48 h later as described in *Materials and Methods*. (**E**) Equal amounts of Ba/F3-gp130-IL-12Rβ1-IL-12Rβ2 cells were incubated with 50% conditioned supernatant containing p40_C197A_ with or without 2 or 4 µg/ml p35_bac/C92A_ for 15 min. Phosphorylation of STAT1 and STAT3 was determined per Western blotting. Total amounts of STAT1 and STAT3 were visualized as internal loading control. The data shown are representative of two different experiments with similar outcomes.

The cDNAs coding for mature murine p35 as well as p35_C92A_ were subcloned into the *E.coli* expression plasmid pet23a. p35 and p35_C92A_ were expressed in *E.coli* as inclusion bodies. After refolding, purification of monomeric p35_bac_ and p35_bac/C92A_ was completed by size-exclusion chromatography (Figure 6A). Final yields of pure, monomeric p35_bac_ and p35_bac/C92A_ of about 15 and 35 µg/liter bacterial culture were obtained (Figure 6B).

Biological activity of pure p35_bac_ and p35_bac/C92A_ was determined using Ba/F3-gp130-IL-12Rβ1-IL-12-Rβ2 cells. As shown in Figure 6C, only monomeric but not multimeric p35_bac_ induced cellular proliferation of Ba/F3-gp130-IL-12Rβ1-IL-12-Rβ2 cells when combined with supernatant containing p40_C197A_. Ba/F3-gp130-IL-12Rβ1-IL-12-Rβ2 cells proliferated with recombinant p35_bac_ and p35_bac/C92A_ supplemented with constant amounts of p40_C197A_ conditioned supernatant from HEK293 cells. As negative controls, p35_bac_ and p35_bac/C92A_ or p40 alone did not induce proliferation of Ba/F3-gp130-IL-12Rβ1-IL-12-Rβ2 cells (Figure 6D). By detection of STAT1 and STAT3 phosphorylation via Western blotting, we verified that reconstituted IL-12 was biologically active. Here, Ba/F3-gp130-IL-12Rβ1-IL-12Rβ2 cells were stimulated with p35_bac_ plus conditioned supernatant containing p40_C197A_. Reconstituted IL-12 specifically induced STAT1 and STAT3 phosphorylation (Figure 6E). Taken together, our data show that p35_bac_ and p35_bac/C92A_ were biologically active and formed IL-12 in combination with p40_C197A_. The biological activity of IL-12 reconstituted with bacterially expressed p35 was, however, more than 100fold less active compared to IL-12.

### Heterodimeric IL-12 induces IFN-γ secretion in primary CD4^+^ T cells

**Figure 7:**
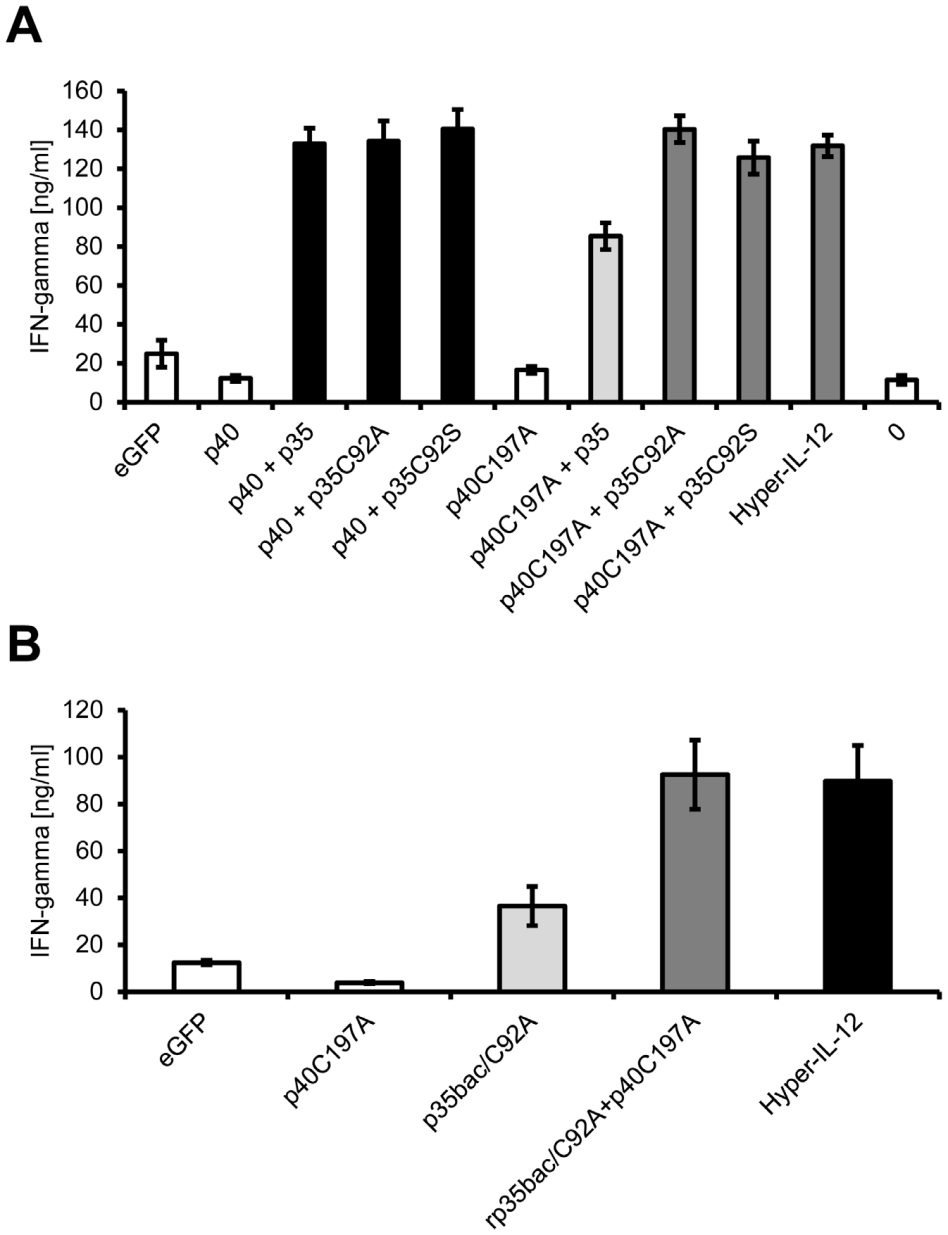
IL-12 induces the expression of Interferon-γ in murine T cells. (**A**) Murine CD4+ T cells were enriched by positive selection from single cell suspended splenocytes and lymphnode cells. 1×10^5^ cells per well were cultured on plates coated with 0.5 µg/ml anti-CD3 and 2 µg/ml soluble anti-CD28. 10% of the indicated cell culture supernatant was added and supernatants were harvested after three days. Secretion of IFN-γ was determined via ELISA. (**B**) The experiment was performed as described under panel (A). Where indicated, 2 µg/ml p35_bac/C92A_ were added. The experiments were performed with at least three individual mice, and data from one representative animal are shown.

We have generated IL-12 by three different approaches. First, we have fused p40 and p35 via a peptide linker (Hyper-IL-12), which we expressed in HEK293 cells. Second, we have transfected HEK293 cells with cDNAs individually coding for the IL-12 subunits p40 and p35. Last, we have purified and refolded p35 expressed in E. coli, which in combination with p40-containing supernatant from HEK293 cells gave rise to IL-12. All kinds of IL-12 were able to induce proliferation of Ba/F3-gp130-IL-12Rβ1-IL-12Rβ2 cells and phosphorylation of the transcription factor STAT3. To have a second physiological readout of IL-12, we isolated CD4^+^ T cells from mice, activated them with α-CD3/α-CD28 antibodies and stimulated them with supernatant from transfected HEK293 cells. As shown in Figure 7A, supernatant containing p40/p35, p40/p35_C92A_ and p40/p35_C92S_ induced the secretion of Interferon-γ (IFN-γ, black bars), whereas supernatant from cells transfected with eGFP or p40 alone did not (white bars). Supernatant containing Hyper-IL-12, p40_C197A_/p35_C92A_ or p40_C197A_/p35_C92S_ induced IFN-γ as well (dark gray bars), whereas supernatant from cells transfected with p40 _C197A_ alone did not (white bar). Supernatant containing p40_C197A_/p35, which only induced a weak proliferative response and little STAT3 phosphorylation (Figure 5D, E), induced IFN-γ secretion, although to a lesser extent than the other p40/p35 combinations (Figure 7A, light grey bar), underlining the reduced capacity of p40_C197A_/p35 to form biologically active IL-12.

Finally, we asked whether bacterial produced p35 was able to induce IFN-γ secretion when combined with p40 _C197A_. As shown in Figure 7B, supernatant from eGFP or p40_C197A_ transfected cells induced little IFN-γ secretion (white bars). Addition of recombinant p35_bac/C92A_ induced IFN-γ secretion on its own (light gray bar). However, combination of p35_bac/C92A_ together with supernatant containing p40_C197A_ drastically increased IFN-γ production (dark gray bar), which was comparable to cells stimulated with Hyper-IL-12 containing supernatant (black bar).

In conclusion, we could show that the three differently produced forms of IL-12 are equally well able to induce IFN-γ secretion from primary T cells, a hallmark of IL-12 activity.

### p35_bac_ fails to form biological active IL-35

**Figure 8:**
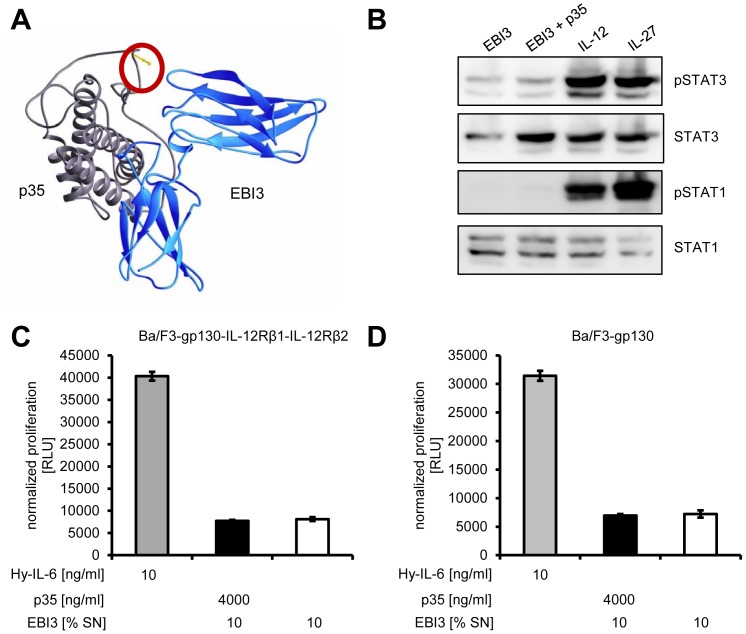
IL-35, formed by recombinant p35, does not induce proliferation or STAT phosphorylation in Ba/F3-gp130-IL-12Rβ1-IL-12Rβ2 cells. (**A**) Schematic representation of IL-35 comprising of p35 (gray) and EBI3 (blue). C92 of p35, which is not connected via a disulfide bond with EBI3, is highlighted with a red circle. (**B**) Equal amounts of Ba/F3-gp130-IL-12Rβ1-IL-12Rβ2 cells were incubated with 50% conditioned supernatant containing EBI3 with or without 2 µg/ml p35_bac/C92A_ for 15 min. Phosphorylation of STAT1 and STAT3 was determined per Western blotting. Total amounts of STAT1 and STAT3 were visualized as internal loading control. (**C**) Equal amounts of Ba/F3-gp130-IL-12Rβ1-IL-12Rβ2 cells were incubated with 10 ng/ml Hyper-IL-6 or 10% conditioned supernatant containing EBI3, either with or without 4 µg/ml recombinant p35_bac_. Cellular proliferation was determined 48 h later as described in *Materials and Methods*. (**D**) The experiment was performed as described under panel (C), but with Ba/F3-gp130 instead of Ba/F3-gp130-IL-12Rβ1-IL-12Rβ2 cells. Cellular proliferation was determined 48 h later as described in *Materials and Methods*. The Western blots shown as well as the proliferation assays are representative of three different experiments with similar outcomes.

After the successful generation of IL-12 *in vitro* by our approach, we finally asked if *in vitro* reconstituted IL-35 was also biologically active and able to induce STAT phosphorylation. Since IL-35 is not stabilized through a disulfide bridge, p35_bac/C92A_ should in principle be able to form active IL-35 with EBI3 (Figure 8A). Unfortunately, we could not detect phosphorylation of STAT1 or STAT3 when Ba/F3-gp130/IL-12Rβ1/IL-12Rβ2 cells were stimulated with IL-35 (Figure 8B). As control, stimulation of Ba/F3-gp130-IL-12Rβ1-IL-12Rβ2 with Hyper-IL-12 and IL-27 lead to robust STAT1 and STAT3 phosphorylation. Furthermore, *in vitro* reconstituted IL-35 did not induce proliferation of Ba/F3-gp130-IL-12Rβ1-IL-12Rβ2 or Ba/F3-gp130 cells (Figure 8C, D). In conclusion, we describe a novel way to produce biologically active IL-12 via bacterially expressed p35, but fail to create IL-35 by this approach.

## Discussion

Our present study has three major findings. We describe for the first time a protocol for expression, purification and refolding of p35 from *E.coli*. In combination with p40, bacterially expressed p35 formed biologically active IL-12. Second, we identified the critical amino acid that links p40 monomers to form the IL-12/IL-23 inhibitor p80. Third, we show that bacterially expressed p35 was not able to form biologically active IL-35.

IL-12 (p35/p40) is stabilized by an inter-chain disulfide bond, linking p35_C92_ and p40_C197_. We mutated both cysteins to alanine, which did not prevent IL-12 formation, but reduced its biological activity compared to wildtype IL-12. IL-12 signals via the receptor combination IL-12Rβ1 and IL-12Rβ2 [8]. We have previously shown that EBI3 in combination with recombinant p28 expressed in *E.coli* formed biologically active IL-27, showing that this is a valid approach to create composite cytokines [17]. We therefore produced recombinant p35_bac_ and the mutant p35_bac/C92A_ in *E.coli* and combined them analogous with supernatant containing p40_C197A_. Biologically active IL-12 was formed, as demonstrated by cytokine-dependent proliferation of Ba/F3-gp130-IL-12Rβ1-IL-12-Rβ2 cells as well as phosphorylation of STAT1 and STAT3. It has to be noted, that bacterially expressed p35 was more than 100 fold less effective than recombinant Hyper-IL-12. In detail, at least 1 µg/ml bacterially expressed p35 in combination with p40 was needed to induce IL-12-dependent proliferation of Ba/F3-gp130-IL-12Rβ1-IL-12-Rβ2 cells. We hypothesize that this was due to ineffective refolding of p35 from inclusion bodies. Only about 20 µg monomeric p35 was refolded from 1 l bacterial culture. The minimal amounts of p35 prevented the characterization of p35 via e.g. circular dichroism (CD)-spectroscopy. It might be speculated that only a small percentage of the pure monomeric p35 fraction were correctly refolded and biologically active, whereas the majority was misfolded or partly unfolded but soluble and biologically inactive. Irrespective of the low biological activity of the refolded p35, we concluded that EBI3 as well as p35_bac_ were functional.

It was previously shown that mutation of C92 to serine within p35 did not prevent IL-12 formation and function, suggesting that the disulfide bond between p40 and p35 mediates stability, but is not needed for IL-12 activity [28]. We verified these results by introducing the C92S and a C92A point mutation in p35, which both were biologically active. Furthermore, we mutated the respective cysteine 192 in p40, which has not been done before. Co-expression of p40_C197A_ and p35_C92A_ in HEK293 cells gave rise to biologically active IL-12, albeit with lower activity compared to wildtype p35 and p40. Interestingly, p40 can be found *in vivo* as disulfide-linked p80 homodimer, and this makes up to one third of the total amount of p40 [7]. We show for the first time that Cys197 of p40 is needed for p80 formation, as p40_C197A_ is only found as a monomer. This means that p40 can either form IL-12 within the ER with p35 via p40_C197_-p35 _C92_, or p80 via p40_C197_-p40_C197_.

One hallmark of IL-6-type cytokines is their ability to signal via the membrane-bound β-receptor gp130. Members of this family recruit specific, in part overlapping, gp130 homo- and heterodimers [1]. In all known cases, this leads to activation of certain downstream signaling cascades, whereas the Jak/STAT-pathway seems to be the major one. Among the seven known STAT proteins, STAT1 and STAT3 (and to a lesser degree STAT5) are phosphorylated after IL-6-type cytokine activation. The degree of STAT-activation between the individual cytokines seems to vary, as IL-27 predominantly activates STAT1 over STAT3. Nevertheless, it has been clearly demonstrated that in principal all IL-6 and IL-12 family cytokines activate the same pattern of STAT proteins. The only known exception to date is IL-35, which can signal via a gp130 homodimer, but solely induces phosphorylation of STAT1, not STAT3. In contrast, IL-6, IL-11 or Hyper-IL-6, which also recruit a gp130 homodimer, induce phosphorylation of both STAT1 and STAT3. To date, this finding is unique for IL-35, as it has not been seen by any other IL-6 type-cytokine. A molecular mechanism that explains this interesting finding is still missing.

Receptor plasticity is a well-documented phenomenon for IL-6-type cytokines [1]. One example is CNTF which usually binds to the non-signaling CNTFRα and recruits a β-receptor heterodimer of gp130/LIFR [29]. Additionally, CNTF can use the IL-6R as alpha-receptor, and this complex also engages gp130/LIFR for signal transduction, showing the use of different α-receptors by the same cytokine [30]. Interestingly, plasticity is not limited to the level of the α-receptor. Oncostatin M (OSM), another IL-6-type cytokine, does not need an α-receptor for signaling, but in contrast directly activates a β-receptor heterodimer of either gp130/LIFR or gp130/OSMR. Since OSM is the only known cytokine that signals through OSMR, the distinct expression of either OSMR makes a cell responsive to OSM alone, whereas LIFR expression allows signal initiation by other cytokines, including OSM, LIF and CT-1 [1]. Another example is IL-30 (IL-27p28), which can signal in combination with EBI3 as IL-27 via gp130/WSX-1, but has been shown to have signaling capacities on its own, as either in combination with cytokine-like factor (CLF) or alone is able to initiate signaling via the IL-6R [17], [31], [32]. In contrast to IL-27, IL-30/IL-6R recruits a gp130 homodimer, and revealed plasticity on the level of the α- as well as the β-receptor [17].

IL-35 has been shown to engage four different β-receptor complexes [10], [12]. Homodimerization of gp130 without the need of a membrane-bound α-receptor has besides IL-35 been seen by viral IL-6 [33] or IL-6 in complex with the soluble IL-6R [16], [34]. However, in both cases phosphorylation of STAT1 and STAT3 was detected, whereas IL-35 solely activates STAT1 [10]. This finding suggests a different, yet unsolved mechanism by which IL-35 engages gp130 homodimerization, that is clearly different by all other known cytokines like IL-6, IL-11, IL-30, Hyper-IL-6 or viral IL-6.

However, we were not able to verify this specific STAT1 activation by IL-35. Hyper-IL-35 was the only cytokine tested that was not secreted from cells. Furthermore, unlinked EBI3/p35 was not detected in the cell supernatant. From our experiments we concluded that EBI3 (in combination with p28_bac_ [17]) and p35_bac_ in combination with p40 were correctly folded and biologically active. However, when p35 and EBI3 were combined, for unknown reasons no active IL-35 was formed. One possibility is that IL-35 needs additional, yet unidentified factors, for efficient biological activity and secretion that were not included in our assays.

Receptor plasticity and cross-talk within the IL-6 and IL-12 families complicates the functional investigation and assignment of individual cytokines, for example in knock-out models. Bacterial expression and refolding of specific cytokines or cytokine subunits especially of poorly secreted species as demonstrated here for p35 can help to circumvent these limitations and thus enhance the understanding of these cytokines.

In summary, our study shows novel aspects in IL-12 biology and highlights that IL-35 differs in several aspects from the other IL-6/IL-12 family cytokines.
